# Glucose Transport and Utilization in the Hippocampus: From Neurophysiology to Diabetes-Related Development of Dementia

**DOI:** 10.3390/ijms242216480

**Published:** 2023-11-18

**Authors:** Caio Yogi Yonamine, Maria Luiza Estimo Michalani, Rafael Junges Moreira, Ubiratan Fabres Machado

**Affiliations:** 1 Novo Nordisk Foundation Center for Basic Metabolic Research, Faculty of Health and Medical Sciences, University of Copenhagen, DK-2200 Copenhagen, Denmark; caio.yonamine@sund.ku.dk; 2 Department of Physiology and Biophysics, Institute of Biomedical Sciences, University of Sao Paulo, Sao Paulo 05508-000, Brazil; maria.michalani@usp.br (M.L.E.M.); rafaeljungesmoreira@gmail.com (R.J.M.)

**Keywords:** Alzheimer’s disease, obesity, cognitive dysfunction, glucose transporters, insulin resistance

## Abstract

The association of diabetes with cognitive dysfunction has at least 60 years of history, which started with the observation that children with type 1 diabetes mellitus (T1D), who had recurrent episodes of hypoglycemia and consequently low glucose supply to the brain, showed a deficit of cognitive capacity. Later, the growing incidence of type 2 diabetes mellitus (T2D) and dementia in aged populations revealed their high association, in which a reduced neuronal glucose supply has also been considered as a key mechanism, despite hyperglycemia. Here, we discuss the role of glucose in neuronal functioning/preservation, and how peripheral blood glucose accesses the neuronal intracellular compartment, including the exquisite glucose flux across the blood–brain barrier (BBB) and the complex network of glucose transporters, in dementia-related areas such as the hippocampus. In addition, insulin resistance-induced abnormalities in the hippocampus of obese/T2D patients, such as inflammatory stress, oxidative stress, and mitochondrial stress, increased generation of advanced glycated end products and BBB dysfunction, as well as their association with dementia/Alzheimer’s disease, are addressed. Finally, we discuss how these abnormalities are accompained by the reduction in the expression and translocation of the high capacity insulin-sensitive glucose transporter GLUT4 in hippocampal neurons, which leads to neurocytoglycopenia and eventually to cognitive dysfunction. This knowledge should further encourage investigations into the beneficial effects of promising therapeutic approaches which could improve central insulin sensitivity and GLUT4 expression, to fight diabetes-related cognitive dysfunctions.

## 1. Introduction

Neurocognitive disorders (dementia) are insidious and progressive diseases, the main symptoms of which include a decline from a previously attained level of cognitive functioning, compromising social and/or occupational functioning [1]. Dementia includes several disorders with different clinical characteristics and etiologies; among the various subtypes of dementia, Alzheimer’s disease (AD) is the most common [1,2,3]. Vascular neurocognitive disorder (vascular dementia, VD) is the second most common cause of dementia and is frequently present in combination with AD [2].

From a molecular point of view, AD has been characterized by the presence of an abnormal accumulation of amyloid beta (AB) and tau protein, together with neurodegeneration [4]. Since neurodegeneration markers are not considered to be specific of AD, AB and tau proteinopathies have been used to define AD among other dementia-related diseases [5]. For more than four decades, it has been described that senile plaques are composed of AB accumulation [6], which induces the tau pathology, characterized by neurofibrillary tangles (NFTs) containing bundles of helical filaments of the microtubule-associated protein tau [7]. Because of that, the “amyloid cascade hypothesis” has been the mainstream explanation for the pathogenesis of AD development [8], which has continued for almost 30 years. However, recent advances in amyloid imaging revealed that amyloid deposits can be seen in many normal subjects, and several AD patients show very few deposits; indeed, trials for AD treatment with drugs targeting AB have failed to fight the disease (for a review, see [9,10]).

On the other hand, tauopathy, which has been related to impairments of amyloid beta precursor protein (APP) metabolism, leading to the accumulation of APP C-terminal fragments, has been staged according to Braak and Braak [11], and its accumulation spreads progressively, correlating with the extension of cognitive and clinical symptoms of AD [9]. Thus, it has been considered that the main factor causing the progression of AD is tau, not AB [9]. However, the description that: (1) synapse loss and microglial activation anteceded NTFs in a mouse model of tauopathy [12], (2) synaptic and memory impairments are not mediated by NFTs [13], and (3) insoluble deposition of tau may be a compensatory protective mechanism [13], compromise the proposal of tauopathy as the main player in AD pathogenesis [13]. Despite the above, abnormal cerebrospinal fluid (CSF) amyloid beta and tau biomarkers have been used, together with some neurodegeneration markers, grey matter volume, and positron emission tomography (PET) scans with 2-[^18^F]fluoro-2-deoxy-D-glucose (2-FDG) uptake, to evaluate the Alzheimer’s continuum [4].

Since AB and tau proteins have lost their central role in the genesis/progression of AD, and considering the advancement of knowledge, new players have been proposed for the pathogenesis of dementia/AD. Indeed, a metabolic hypothesis has emerged, based on the observation of altered regional cerebral glucose utilization association with dementia/AD [14,15,16]. Since then, mechanisms related to neuronal glucose utilization have assumed extreme relevance in the pathophysiology of AD, and diabetes mellitus (DM) has become an important player in the pathogenesis of AD [17].

## 2. The Role of Glucose in Neuronal Function

Glucose is a fundamental fuel substrate for the proper development and functioning of the brain; moreover, the creation of new brain structures and connections is the most expensive energetic effort of the energy production of the whole body [18]. In healthy adult humans, in the post-absorptive state, the brain is responsible for ~50% of total body glucose use, despite accounting for only 2% of the total body mass [19]; this evinces the relevance of glucose for the functioning and maintenance of the brain. In terms of total-brain glucose consumption, the extent of the glucose requirement for neuronal metabolism is still unclear, not only because of the consumption by other cells, but also because glucose may be primarily metabolized by astrocytes to lactate, which can be exported and eventually used as fuel by neurons [20,21]. Nevertheless, neurons take up and metabolize glucose through glycolysis as the primary fuel for normal function (for a review, see [12,20,21]).

The fundamental role of glucose for neuronal development and functioning has been demonstrated for decades, but it remains a matter of concern in several investigations, mainly because of the particularities of different neuron types and brain areas. However, on the whole, it is clear that low glucose concentrations induce brain damage, including neuron loss, both in vivo and in vitro. In vivo, reduction in the cerebral glucose supply has been associated with neuroglycopenic symptoms, including permanent impairment in mental status with neuronal loss; these events may be caused by recurrent peripheral blood glucose lowering [22,23], or the restriction of glucose disposal to the cerebral interstitium [24,25]. In vitro, a low glucose concentration (0–0.25 mM) is known to induce apoptosis in SH-SY5Y cells, in a concentration- and time-dependent manner [26].

On the one hand, the effects of low glucose concentration upon brain/neuron functioning and preservation seem to be clearly established; on the other, the effects of high glucose concentration are still very poorly understood, despite their relevant role. In vitro, 24-h SH-SY5Y cell viability seemed to progressively increase in response to glucose concentrations from 5.6 to 7.0 mM, decreasing drastically in concentrations over 7.8 mM [27]. Similarly, although a 24-h culture of SH-SY5Y with 8.3 to 14.5 mM glucose had no effect on cell viability, the 48-h culture revealed a clear glucose concentration-dependent reduction in cell viability; moreover, the addition of insulin (40 uU/mL) promoted a further reduction in cell viability in both 24-h and 48-h cultures [28]. In addition, a very high concentration of glucose (30.5 mM) increased the expression of inflammatory cytokines in astrocytes and also the susceptibility of undifferentiated SH-SY5Y cells to injury by oxidative stress; however, the participation of hyperosmolarity in the observed effects was not clearly analyzed [29].

Additionally, in vivo effects of high glucose concentrations are even less known. Obviously, DM is the first example we think of; however, this syndrome involves a myriad of different conditions, including: (1) varying levels of plasma glucose, insulin, and other metabolic or hormonal substrates (such as lipids, proteins, glucagon, and cortisol, etc.), (2) a varied production of advanced glycated end products (AGEs), (3) different grades of oxidative, inflammatory, and endoplasmic reticulum stress, and (4) the time of evolution of the disease. Considering this, it becomes clear that the in vivo effects of hyperglycemia must be carefully analyzed in DM-related diseases; at least, type 1 DM (T1D) and type 2 DM (T2D) should be separately evaluated. Moreover, although T1D definition is based on primary autoimmune beta cell destruction, usually leading to absolute insulin deficiency [30], its treatment frequently imposes a hyperinsulinemic state, which can course with recurrent episodes of hypoglycemia and insulin resistance as well.

Considering the above, some data should be emphasized. In the brain of healthy humans, increased plasma glucose concentrations (during a four-hour hyperglycemic clamp) rapidly increases the intracerebral glucose (10 min) and fructose (20 min) concentrations (measured by ^1^H magnetic resonance spectroscopy scanning), remaining during the four-hour clamp and activating the polyol pathway [31]. In T2D subjects, chronic hyperglycemia leads to a parallel increase in CSF glucose and polyol concentrations, indicating that the neurotoxic polyol pathway (glucose → sorbitol → fructose) is activated [32]. Additionally, young adults at high risk of developing obesity (presenting increased body weight, body mass index, body fat mass, plasma glucose, and plasma insulin) show a brain glucose uptake (measured by [^18^F]FDG) higher than that of subjects at low risk [33]. Whether these metabolic changes can chronically induce impaired neuronal functioning and neurodegeneration remains unclear.

## 3. Glucose Supply to the Brain and Glucose Transporters

Once the importance of glucose for brain/neuron functioning has been highlighted, it becomes essential to understand the mechanisms by which glucose is offered to and taken up by neurons. Since glucose is a polar molecule, insoluble in the plasma membrane (PM) and in any other intracellular membrane, glucose transporter proteins are essential for all glucose fluxes in the body [34,35,36]. Because of this, the glucose supply to cerebral neurons can only be understood by considering the expression of the different glucose transporters in each cell type and area of the brain.

### 3.1. Glucose Transporters

Glucose transporters started to be characterized early in the 1990s. The ones responsible for the facilitative transport of glucose downhill its gradient of concentration were designated as GLUTs (facilitative glucose transporters), and the ones carrying glucose against its gradient of concentration, coupled to sodium transport, were designated as SGLTs (sodium/glucose cotransporters). GLUTs are expressed in all cell types to supply glucose for the cell metabolism and function, whereas SGLTs are classically expressed in the epithelium (mainly in the gut and kidneys), ensuring the uptake of all luminal glucose against its gradient of concentration [36,37]. Moreover, the genes responsible for expressing those proteins belong to the *SLC* (solute carrier) family, and two families were related to glucose transporters: *SLC2A*, which codifies GLUTs, and *SLC5A*, which codifies SGLTs [37]. The names of both of the proteins and genes (as well as their symbols) must be further followed by the number of the isoform. Nowadays, there are fourteen isoforms of GLUTs and five of SGLTs, showing a variable tissue/distribution; however, several of those isoforms have not been significantly related to important glucose fluxes in the body [35,36,38].

GLUTs and SGLTs differ in their kinetic properties, which are depicted by their Km. A low Km implies a high affinity for the substrate, but a low transport capacity (saturates at low glucose concentration), whereas a high Km represents the opposite [34,35,38]. The Km of each transporter can vary according to the conditions of transport measurement, the tracer utilized, and the cell type, among other variables [38,39], and some spurious Km values have been reported elsewhere. In general, it is well accepted that most of the isoforms have a low (1–2 mM) Km, GLUT4 has an intermediary (~4 mM) Km, and GLUT2 has a high (~17 mM) Km [38,39]. Variations have also been observed in the Km of cotransporters SGLT1 (~0.5 mM) and SGLT2 (~5 mM) [39]. To understand the true role of each transporter in cellular glucose uptake, these kinetic properties need to be analyzed considering the extracellular concentration of glucose. As to the physiological range of blood glucose (4 to 8 mM): (1) low Km transporters work under saturated conditions, even at the lowest concentrations; (2) the high Km transporter GLUT2 does not transport glucose at low glucose concentrations, starting glucose transport only at concentrations above ~6 mM, but can increase its transport capacity up to very high concentrations; and (3) the intermediary Km transporter GLUT4 (~4 mM) can vary its transport capacity parallel to physiological variations in blood glucose concentrations [35,38,40]. Additionally, as blood glucose increases (over 5–6 mM), intracellular GLUT4 is translocated to the plasma membrane in response to insulin, thereby increasing glucose transport significantly [35,38,40].

Frequently, more than one isoform expresses in the same cell, and in this case, the affinity/capacity of each isoform must be considered to determine the relevance of the isoform for the glucose supply in that cell. In the brain, several GLUTs and two SGLTs have been described, making the understanding of the cerebral glucose supply/utilization very complex, as will be discussed below.

### 3.2. Glucose Transport and Transporters in the Blood–Brain Barrier

Most of the cerebral blood irrigation system is constituted by the blood–brain barrier (BBB), which consists of endothelial cells connected by tight junctions between the cells, thus devoid of fenestrations. This prevents the free entry of blood-derived substances (mainly hydrophilic molecules) into the cerebral interstitium, and preserves the proper composition of the neuronal environment for perfect cell functioning and maintenance [41]. Glucose is a polar molecule that leaves the peripheral blood via the endothelial fenestrations; thus, the glucose flux across the BBB operates through a transepithelial transport, which partially limits the entrance of glucose into the cerebral parenchyma [20].

GLUT1, the first glucose transporter to be described, has been reported to have a ubiquitous distribution in tissues/cells and is the only isoform expressed in endothelial cells [34,37]. Because of that, it is the only passageway for glucose in the BBB, regulating the availability of the substrate to the cerebral interstitium [20]. GLUT1 has a low Km for glucose transport [37]; therefore, it is considered as operating under a saturated condition in the physiological range of glycemia, and variations in its transport capacity can only be achieved by regulating the quantity of the transporter.

Interestingly, GLUT1 has been described as presenting an asymmetric distribution in the BBB; however, the literature is considerably confusing regarding this distribution [42]. In rats, mice, and rabbits, the abluminal:luminal ratio has been described as up to 4:1. However, the same does not occur in humans, in whom two types of endothelial cells are observed: the high-GLUT1-expressing type A cell exhibiting a higher density of abluminal membrane GLUT1, and the low-GLUT1-expressing type B cell exhibiting a higher density of luminal membrane GLUT1; this way, two types of capillaries can be found, and also, along the same capillary, the type of cell may change. Considering this, it is clear that the BBB glucose transport can be regulated not only by altering the total amount of GLUT1, but also by altering its bimodal distribution pattern (for a review, see [43]). Teleologically, we might suppose that the higher density of GLUT1 in the abluminal membrane would favor the efflux of glucose to the interstitium, but that is far from being confirmed. For instance, increased expression of the BBB GLUT1, mainly at the luminal membrane, has been associated with increased glucose flux across the BBB in seizures [43]. Finally, it is reasonable to suppose that chronic hyper- or hypo-glycemia could modulate the expression of GLUT1 and its distribution in the BBB, but, again, that is not clearly demonstrated, especially in humans.

Despite all doubts concerning the expression and distribution of GLUT1 in the human BBB, the fact is that an important restriction in glucose passage occurs. It has been described that the median glucose concentration in the CSF (the same as that of the brain interstitium) is approximately 60% of that in the blood, at normal glycemia levels [44]. This CSF/blood glucose ratio of ~0.6 has recently been confirmed, taking into account hourly blood glucose measurements during a six-hour period before lumbar puncture [45]. However, conflicting data have been reported regarding the 0.6 CSF/blood glucose ratio as blood glucose increases. While in healthy and T2D monkeys an unchanged CSF/blood glucose ratio was reported, in healthy and T2D humans the ratio seems to negatively correlate with blood glucose, indicating that the glucose flux across the BBB does not increase in parallel with blood glucose [44,45].

Finally, it is necessary to comment on fact that the expression of SGLT1 and SGLT2 in the endothelium of the BBB has been proposed elsewhere [46,47,48,49,50,51,52,53,54]; however, all of those studies present several methodological problems, which invalidate the assertion above. Indeed, very well conducted investigations using PET scans with α-methyl-4-[18F]fluoro-4-deoxy-D-glucopyranoside (Me-4FDG), a specific substrate for SGLT transporters, demonstrate that Me-4FDG did not enter the brain, definitely excluding the presence of SGLTs in the BBB of rats and humans [55,56,57].

### 3.3. Glucose Transport and Transporters in the Circumventricular Organs (CVOs)

CVOs have unusually dense and permeable capillary networks that facilitate the movement of substances from the blood to the interstitium and vice versa [58], unlike other nervous system areas wherein the BBB limits the permeability of solutes such as glucose and hormones. The hallmarks of CVOs are fenestrated capillaries (like in the peripheral endothelium), high permeability, small-sized areas, and location around the third and fourth ventricles [59]. CVOs include the organum vasculosum of the lamina terminals, the median eminence and adjacent neurohypophysis, the area postrema, and the subfornical organ (for a review, see [60]). In these areas, several functions are modulated in response to rapid variations in peripheral glycemia, and neurons in these areas—in addition to the neuronal typical GLUT3 transporter—express GLUT2 (high Km for glucose), providing a glucose sensing function as in the pancreatic beta cell [61]. Central GLUT2-dependent glucose sensing is deeply related to the control of feeding and thermoregulation in these areas, among several other functions (for a review, see [62]). This issue will not be explored here since it is beyond the scope of the present review.

### 3.4. Glucose Transport and Transporters in the Hippocampus

After considering the glucose transport in the BBB (Section 3.2), and before specifically discussing the glucose influx into hippocampal neurons, we must point out the important role that astrocytes play in the brain. Through GLUT1, astrocytes (and other glial cells) take up and metabolize glucose; thus, releasing glycolytically-derived lactate to neurons, which is an additional important fuel source [20,21]. Moreover, GLUT2 has also been reported to be expressed in astrocytes, but its function is unclear, pointing out that, at low interstitial glucose concentrations, GLUT2 cannot bind/transport glucose [42,63].

GLUT3 has been referred to as the neuron-specific glucose transporter [34]. It is largely observed in the brain, mainly in neuropil segments, and has been considered as fundamental for neuronal glucose supply [20]. It displays a very low Km for glucose [37]; because of this, it works in a saturated way at physiological glucose levels, even considering the restriction of the BBB to glucose disposal. On the other hand, its low Km is accompanied by a high kinetic affinity, which guarantees glucose binding/transport even when glucose concentration drops sharply in the cerebral parenchyma [35].

It was reported that insulin increases PM-associated GLUT3 in rat hippocampal neurons, although insulin neither promoted the fusion of GLUT3 with the PM nor increased neuronal glucose uptake; despite these clear results, this study was published under the title “Insulin regulates neuronal glucose uptake by promoting translocation of glucose transporter GLUT3” [64]. At the same time, in the human-derived neuronal cell line SH-SY5Y, it was clearly demonstrated that PM GLUT3 does not increase in response to insulin as GLUT4 does [65]. However, more recently, GLUT3 translocation has been described again in the hippocampus of 3xTg-AD mice [66], but this report has some methodological problems such as: (1) no information regarding the insulin stimulus applied; (2) no information regarding the number of animals used for statistical analysis; (3) GAPDH was inappropriately used for normalization of the results in the plasma membrane (this protein is not bound in the PM); (4) insulin did not increase PM GLUT3 in control mice; and (5) GLUT3 content, actually, decreased in the PM of insulin-stimulated 3xTg-AD mice. Thus, if the result is true, its correct interpretation would be that insulin promotes the internalization of GLUT3, rather than the reduction of GLUT3 translocation, as highlighted in the title of the manuscript [66]. In sum, GLUT3 is a low-capacity glucose transporter expressed in the PM of neuropil segments, guaranteeing basal glucose support to the neuronal activity.

Early in the 1990s, other GLUTs were also described in neurons, and thus, an important history for the classic insulin-sensitive GLUT4 in murine brains started to be constructed. GLUT4 was first reported in the medulla oblongata and hypothalamus [67,68,69], then in the cerebellum [70,71], and finally in the dentate gyrus of the hippocampus [72]. Later on, it was described that the GLUT4 protein translocates to the plasma membrane in response to insulin, both in rat hippocampal neurons [73] and in the human neuronal cell line SH-SY5Y [65], exactly as it does in adipose and muscle tissues. Moreover, like in skeletal muscle cells, where GLUT4 is also translocated by muscle contractions, in rat primary hippocampal neurons the action potential firing also triggers the insertion of GLUT4 into the plasma membrane, especially in the axonal nerve terminals [69]. These data ascribe the hippocampal GLUT4 protein the same functional role it plays in the classic insulin-sensitive tissues [40], placing the transporter as a key player in the hippocampal metabolic glucose utilization. Additionally, we and others have already described GLUT4 in hippocampal neurons of the human brain, where it seems to play an important role under pathological conditions, as further discussed [74,75,76].

The GLUT8 transporter has also been reported in hippocampal neurons, astrocytes, and microglia of murine, but in the intracellular compartment, spreading to other brain areas [20,35,36,37]. GLUT8 was also reported as capable of translocating by insulin in the hippocampus [20]; however, even though GLUT8 is insulin-responsive in blastocysts [37]; this was not confirmed in neurons [37,77,78]. Moreover, GLUT8 displays a very low Km for glucose transport, compromising its conceivable role [35,37]; furthermore, as GLUT8 is only found in intracellular compartments and there is no evidence for any significant intracellular free glucose concentration, its relevance in brain glucose consumption should be disregarded, especially in neurons that express GLUT4.

Low levels of GLUT5 have been detected in astrocytic and microglial cells of murine brains [20,35], and it has already been observed in human hippocampus [75]. However, GLUT5 is considered a fructose transporter, highly expressed in intestinal cells where it plays an important role in the absorption of this substrate [34,35]. Thus, the physiological significance of the extraintestinal expression of GLUT5 at low levels is unclear, especially considering the very low concentration of fructose in the blood (<0.1 mM) compared with its high Km (>10 mM) for glucose transport [35]. Moreover, in addition to the fact that fructose is found at low levels in the blood, it is not clear if this substrate can cross the BBB, where GLUT5 has never been described [35].

SGLT1 was first proposed to be expressed in neurons of the hippocampus and cerebellum of pigs [79], and further confirmed in the hippocampus of rats by both Me-4FDG accumulation and immunohistochemistry [55,80]. Despite that, an important question to be asked is: if brain neurons have an abundant expression of other facilitative glucose transporters, and the influx glucose gradient is constantly preserved, why would it need an energetically very expensive glucose supply via the low-capacity SGLT1?

**Figure 1:**
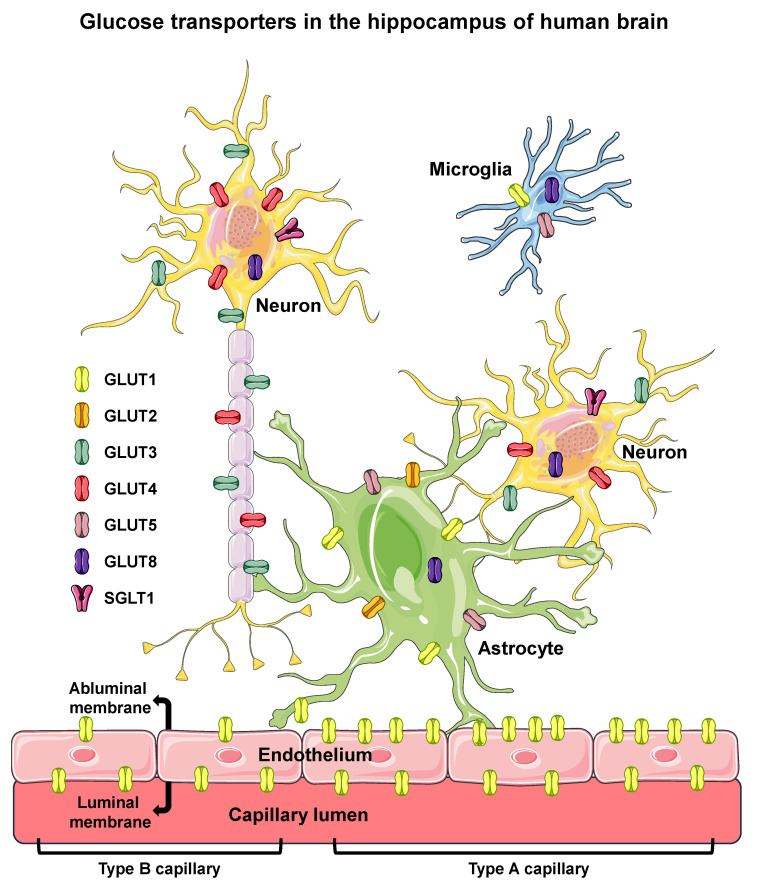
Glucose transporters in the hippocampus of human brain. Glucose transporters are shown in endothelial (of BBB), microglia, astrocyte, and neuron cells of the hippocampus of human brain. Six different isoforms of facilitative glucose transporters (GLUTs) and one isoform of sodium/glucose cotransporter (SGLT1) are shown in the cells in which they were clearly reported to be expressed in humans. The intracellular localization of GLUT8 is depicted since that isoform has not been observed in the PM. In the BBB endothelium, high-GLUT1-expressing type A cells, exhibiting a luminal/abluminal GLUT1 ratio < 1 (on the right), and the low-GLUT1-expressing type B cells, exhibiting a luminal/abluminal GLUT1 ratio > 1 (on the left) are represented, revealing that the two types of endothelial cells can coexist in different segments of the same capillary. BBB, blood–brain barrier; PM, plasma membrane. Names and symbols of the glucose transporters are in accordance with the UNIPROT database (https://www.uniprot.org, accessed on 10 October 2023); parts of the figure were drawn using Servier Medical Art (https://smart.servier.com, accessed on 8 May 2023).

Considering the above, and regarding the participation of distinct isoforms of GLUTs in the supply of glucose to hippocampal neurons, we highlight two key points: (1) the GLUT1-dependent glucose transport across the BBB, providing glucose to the cerebral interstitium, and (2) the GLUT4-dependent glucose influx to neurons. Moreover, like the well-established pivotal role of GLUT4 in peripheral insulin-stimulated glucose uptake, and the relationship with metabolic diseases [40], it is now clear that GLUT4 also plays a fundamental role in the metabolic homeostasis of hippocampal neurons, which can also play a role in diseases related to impaired neuronal homeostasis [81]. Figure 1 summarizes the glucose transporters confirmed to be expressed in the hippocampus.

## 4. Diabetes Mellitus and Cognitive Impairment

### 4.1. DM, Hyperglycemia and Hypoglycemia

To understand pathophysiological mechanisms potentially involved in the relationship between DM and cognitive impairment, we must first point out that although DM is defined as a hyperglycemic disease, iatrogenic recurrent hypoglycemia will occur to a greater or lesser degree in consequence of sulfonylurea, glinide, or insulin treatments (for a review, see [82,83]). Hypoglycemic episodes are higher in T1D individuals, for whom insulin therapy is mandatory, and 30–40% of T1D subjects experience an average of 1–3 episodes of severe hypoglycemia each year. In insulin-treated T2D, this rate is one-third of that in T1D; besides, in both T1D and T2D, the rate of any type of hypoglycemia is ~50-fold higher than those of severe hypoglycemia [80]. The advent of intensive glycemic therapy, to achieve better glycemic control and prevent diabetic complications, has 3-fold increased the risk of hypoglycemia. For instance, older estimates (from the ~1980s) were that 2–4% of T1D subjects died of hypoglycemia, whereas more recent estimates are that 6–10% of T1D die of hypoglycemia [84]. Since the incidence of severe hypoglycemia has increased, obviously the incidence of mild hypoglycemia or even hypoglycemia unawareness must have also increased.

As commented on above (Section 2), a drop in the brain interstitial glucose concentration can induce short- and long-term neuroglycopenic outcomes, including permanent impairment of mental status with neuronal loss. Therefore, when attempting to understand diabetes-related mechanisms involved in cognitive impairment, it must be clear that both recurrent hypoglycemic episodes and long-term hyperglycemia can be involved in the genesis of cognitive impairment. This makes the pathogenesis and/or pathophysiology of the association of DM/AD very intricate, requiring careful analyses.

### 4.2. Brief Hystory of DM and Cognitive Impairement

In the PubMed database, the first reference to the relationship between DM and brain damage was based on the occurrence of “mental disturbs” in DM subjects [85]; ten years later, brain damage was suggested to occur in DM [86], and finally the relationship between DM and AD was associated with recurrent hypoglycemia [87].

On the other hand, in a study of 839 hospital records of various dementia diagnoses, sixty-three cases of DM were found, but none of these in the groups of AD; furthermore, fasting glycemia was lower and the area under the oral glucose tolerance test (OGTT) curve was smaller in the AD group compared with that of control group, suggesting that AD and DM did not coexist [88]. Curiously, the study revealed elevated serum insulin levels during the OGTT in AD subjects, as compared to control subjects [88], but that was not considered as relevant, probably because the concept of insulin resistance (with hyperinsulinemia) was not well established at that time. To make that confusion even worse, Wolf-Klein and colleagues reported that AD subjects were physically healthier, presenting a lower prevalence of several age-related diseases, including diabetes, as compared with age-matched subjects with normal mental status; based on that, the authors suggested a protective effect of hyperglycemia on the brain [89]. However, this study was later clearly challenged by another one [90].

Despite some spurious studies discarding any relatioship between DM and AD, cognitive impairment in DM was becoming increasingly evident, especially in young people who developed diabetes at an early age; all of these studies evinced that recurrent hypoglycemias caused cognitive impairment and brain damage [91,92]. Since then, the concept that neuroglycopenia in DM subjects—resulting from a hypoglycemic episode—leads to neuronal degeneration with cognitive decline and dementia, has been consolidated. That must be considered not only in insulin-treated T1D, but also in several cases of some drug- and insulin-treated T2D (for a review, see [93]).

### 4.3. T2D, Hyperglycemia and Dementia

The 1990s have marked a huge expansion in the incidence/diagnosis of T2D, along with the growing incidence of obesity. At those times, the idea of an association between AD and iatrogenic hypoglycemia in DM had been established; however, the association of T2D (independently of hypoglycemia) and AD was only beginning to be considered, in spite of some serious mistakes. That was clearly evinced in an editorial with a challenging title: “Alzheimer’s disease and non-insulin-dependent diabetes mellitus: common features do not make common bedfellows”, in which the author urged to determine whether the presence of AD or T2D reduced the occurrence of each other [94].

Fortunately, Messier and Ganong [95] solved the riddle in a seminal review, in which they pointed out that there were some studies in animal models and in T2D elderly subjects associating altered glycemic regulation and impairment of learning and memory processing. Therefore, the authors emphasized the importance of determining prospectively whether altered glycemic regulation was linked to a faster progression of AD, just as they raised the proposal of whether AD subjects might benefit from treatments aiming to normalize glycemic control and improve insulin sensitivity [95]. These investigations started with the Rotterdam Study [96], a large population-based study in the elderly, which revealed a positive association between DM and dementia (odds ratio: 1.3); pointing out that this association was strong in insulin-treated DM (odds ratio: 3.2). The association of DM was observed with AD, but it was stronger with vascular dementia [96]. Further, in a prospective study with the Rotterdam cohort, nondemented participants (6370 subjects) were followed up for 2.1 years, and the results demonstrated that DM doubled the risk of dementia (relative risk 1.9) and AD (relative risk 1.9); again, insulin-treated subjects were at highest risk of dementia (relative risk 4.3), concluding that DM contributes to the development of all dementia [97]. Shortly afterward, analyses with magnetic resonance imaging (MRI) revealed that hippocampal and amygdalar atrophy (markers of the degree of AD), assessed in 506 subjects from 60 to 90 years of age, were associated with both T2D and increasing insulin resistance in non-DM subjects, regardless of vascular pathology [98].

In 2010, a meta-analysis study revealed that obesity and DM significantly and independently increase the risk of AD, and proposed that physiological changes common to both obesity and DM predispose to AD [99]. Although this study did not specify the types of DM included, as the authors analyzed obesity alone and along with DM, we can suppose it should be T2D [99]. Soon afterward, Ohara and collaborators started to publish results from the remarkable Hisayama Study, in which a total of 1017 dementia-free subjects aged ≥60 years (who underwent an OGTT) were followed up for 15 years; the results revealed that diabetes is a significant risk of all causes of dementia, AD, and probably VD [100]. Moreover, the data demonstrated that the diseases correlated with elevated two-hour post-load glucose (2-hPG) levels, but not with fasting plasma glucose [100]. Later, the Hisayama Study evaluated the total brain, intracranial, and hippocampal volumes of 1238 subjects (aged ≥65 years) by MRI and the results revealed that a longer duration of DM and elevated 2-hPG levels were risk factors for brain atrophy, particularly for hippocampal atrophy [101].

Since then, metabolic alterations of the T2D syndrome, independently of iatrogenic hypoglycemia, have definitely been accepted as accelerators of cognitive decline [102]. Regarding that, a remarkable consensus review was published by an international group of scientists concluding that T2D is a risk factor for both AD and VD, based on the impairment of the brain glucose utilization, which seems to involve central insulin resistance, triggering the search for the molecular mechanisms involved in this relationship [103]. Later on, the interaction between DM and AD began to be proposed as bidirectional: DM causing neurodegeneration, and AD, in turn, influencing systemic glucose metabolism, with various molecular and clinical factors participating in these interactions [104].

## 5. Pathophysiological Mechanisms Associated to DM and Dementia

Over time, an important factor that has reinforced the DM/AD relationship is the detection of pathophysiological cellular, molecular, and clinical factors which could be common to both diseases [104]. The mechanisms involved in the pathogenesis of hyperglycemia-related cognitive dysfunction included: macro- and micro-vascular disease, dyslipidemia, hypertension, insulin resistance and hyperinsulinemia, direct toxic effect of hyperglycemia on the brain, inflammatory- and oxidative-stress, and advanced glycation end products, among others.

### 5.1. Inflammation and AGEs

In past times, the inflammatory cytokine interleukin-1 beta was proposed as a common cause of AD and DM, which could be related to amyloid beta (AB) deposits found in the brain of AD patients and in the pancreatic beta cells of T2D patients as well [105]. However, we must bear in mind that the fundamental role of AB in the genesis of AD has been increasingly questioned [10]. Despite that, the involvement of inflammation in neurodegenerative processes has been increasingly described [106,107]. Among the inflammatory cytokines, the tumor necrosis factor (TNF) has emerged as an important player in the inflammation-induced AD-related damage because, although TNF enhances synaptic function at physiological levels [108], at increased levels, it can induce deleterious effects, leading to hippocampal dysfunctions [109]. Moreover, in humans with mild to severe AD, increased levels of TNF has been associated with a two-fold increase in the rate of cognitive decline over six months [110]. In addition to TNF, hyperglycemia has been found to increase the expression of IL1, IL4, and IL6 at the neurovascular unit [42], which is followed by the activation of NFKB, a powerful repressor of the GLUT4 transporter, as it will be addressed below.

An increased cellular concentration of glucose, resulting from hyperglycemia, is known to induce oxidative stress, a link for all DM-related complications [111]. Increased glucose metabolism in the glycolytic pathway increases the electron flow in the mitochondrial respiratory chain, and that increases the generation of reactive oxygen species (ROS), especially the superoxide anion [111]. As a consequence, the expression and activity of poly(ADP-ribose) polymerase increases, modifying the structure of glyceraldehyde-3-phosphate dehydrogenase (GAPDH). Thus, an increased glycolytic flow deviates GAPDH and dihydroxyacetone (DHA) to the generation of methylglyoxal (MGO), a highly reactive oxoaldehyde that interacts with proteins, phospholipids, and nucleic acids, leading to the irreversible formation of AGEs [111]. In hyperglycemic states, AGEs can be further increased by the coactivation of the polyol pathway: aldose reductase catalyzes glucose conversion into sorbitol, which is converted into fructose by sorbitol dehydrogenase. Fructose is highly reactive with proteins, leading to the rapid formation of AGEs; in addition, by depleting NADPH, the polyol pathway impairs glutathione resynthesis, favoring oxidative stress that increases the generation of AGEs [111].

DM is known to increase the generation of advanced glycated end products (AGEs), involved in the genesis and progression of several diabetic complications; besides, the interaction of AGEs with their receptor RAGE (advanced glycation end product receptor) can activate pro-inflammatory signals, reinforcing inflammation [112]. The involvement of AGEs in DM-related AD was proposed in the past [113], and recent studies have confirmed that several impaired neurocognitive conditions are accompanied by increased AGE activity and RAGE expression in the hippocampus [114,115]. Hyperglycemia increases the brain interstitial glucose concentration and CSF glucose, and activates the polyol pathway in the brain [32,33]; additionally, fructose (a product of the polyol pathway) is an excellent substrate for the generation of AGEs. In fact, recent advances concerning molecular mechanisms involved in the AGE-induced degenerative complications in DM have revealed the participation of several players in a complex interplay among endoplasmic reticulum, inflammatory, and oxidative stress [116]. Because of that, the involvement of AGEs in DM-related dementia has been repeatedly reported, together with several other cellular injury pathways, which can also trigger/increase degenerative processes in the brain (for a review, see [116,117,118]).

### 5.2. Insulin Resistance (IR) and Obesity

Insulin resistance is an important matter to be considered in the relationship between DM and AD, but it is important to carefully distinguish what peripheral IR is and what central IR is (especially in brain areas related to AD). Firstly, as already discussed here, peripheral IR is a feature of T2D, and seems to evolve from obesity (among other factors) in the same way that T2D evolves; so, it is perfectly plausible that obesity and IR, as well as T2D, contribute to varying degrees to the development of AD, and, perhaps, additively and/or synergistically (for a review, see [99,119,120,121]).

Insulin signaling involves a myriad of pathways and related molecules, which may vary in relevance according to the cell type and the specific insulin effect. The most classic insulin signaling pathway, which regulates the translocation of GLUT4 to the plasma membrane, was characterized early in the 1990s. It involves the binding of insulin to its receptor (a tyrosine kinase), which will phosphorylate the insulin receptor’s substrate proteins (IRS1/2) and recruit adaptors to the PM, such as phosphatidylinositol 3-kinase (PI3K). PI3K increases phosphatidylinositol 3,4,5-trisphosphate (PIP3) at the PM, leading to the activation of protein kinase B (PKB, alias AKT). Later, in the 2000s, the AKT substrate 160 (AS160), a Rab-GTPase activating protein, was characterized as highly insulin-responsive and involved in the translocation of GLUT4-containing vesicles to the PM (for a review, see [40]). Since the characterization of GLUT4 expression in neurons of some brain areas, and its insulin-stimulated PM translocation, it has been expected that all of the steps of insulin signaling related to glucose uptake are also operating in GLUT4-expressing neurons [40]. Thus, an important question began to be asked: is it possible that insulin resistance (IR) also develops in neurons, involving the classic peripheral defects of the pathway related to the regulation of glucose uptake?

In 2005, de la Monte and collaborators drew attention to the fact that human postmortem brains from well characterized AD subjects showed molecular and biochemical alterations typical of T2D-related peripheral IR [122], and that could compromise neuronal energy metabolism, plasticity, and survival [123]. In fact, insulin resistance (IR) together with insulin-like growth factor 1 (IGF1) resistance (IGF1R) have been reported to be installed in neurons from human postmortem hippocampi of AD subjects, involving defects in the signaling cascades following insulin receptor activation [120,123]. Neuronal IR was also observed in neuronally-derived extracellular vesicles extracted from the blood of AD subjects. Further, it was definitely shown that IR/IGF1R in AD brains involve reduced receptor binding and IRS/PI3K/PKB pathway activation, as well as reduced anti-apoptosis-related mechanisms of insulin/IGF1, such as the Bcl2-associated agonist of cell death (BAD), forkhead box protein O1 (FOXO1), glycogen synthase kinase 3 beta (GSK-3B), and NFKB (for a review, see [123]).

In addition to the effect of IR on neuronal glucose utilization, IR has a direct impact on tau- and AB-related activity. IR-induced over-activation of GSK3B promotes the hyper-phosphorylation of tau, with tau misfolding and fibril aggregation; additionally, IR/IGF1R impair the expression of the microtubule-associated protein tau (*MAPT*) gene, further contributing to the tau pathology [123]. IR has also been reported to impair amyloid B precursor protein (ABPP) processing and ABPP/AB clearance; interestingly, the accumulation of ABPP/AB contributes to the impairment of insulin signaling, by reducing the ability of the hormone to bind to its receptor, thus creating a vicious cycle [123]. Regarding brain insulin resistance and dementia, several reviews can be consulted in the literature [33,103,118,120,121,122,123].

Furthermore, the description of GLUT4 expression in neurons from the hippocampus and cerebellum, as well its insulin-induced translocation to the plasma membrane in neurons (see above Section 3.4), has added a new chapter in the discussion of IR/T2D/AD.

In the last forty years, the 2-FDG PET scan has revolutionized studies of brain glucose uptake (and metabolism) in humans with dementia/AD. By using 2-FDG, a substrate for GLUTs, regional glucose accumulation in the brain began to be evaluated [14,15,16]. After being taken up by the cell, 2-FDG is phosphorylated and remains trapped in the cell, proportionally to the glucose phosphorylation rate. That has been referred to as brain glucose utilization (BGU), but extensive literature also considers that as the cerebral metabolic rate of glucose. Moreover, additional considerations should be taken into account, such as the rate of the glucose transport across the BBB and the rate of glucose uptake by cells other than neurons. Despite these concerns, it is well accepted that, in AD, reduced accumulation of 2-FDG is observed in frontal, temporoparietal, and cingulate regions at early stages of the disease.

In an investigation conducted with 749 subjects (81.0% cognitively normal and 20.6% with DM), the 2-FDG AD signature was more common in DM subjects (48.1%) than in non-DM subjects (28.9%), indicating that DM increases AD-related glucose hypometabolism in the brain [119]. However, the AD signature 11C-PiB ([11C]-labeled Pittsburg compound B, a marker for amyloid-beta) accumulation ratio was similar in DM against non-DM subjects. Moreover, in non-DM subjects, a one percent increase in HbA1c (glycated hemoglobin A1c) was associated with glucose hypometabolism in cognitively normal subjects [119]. All these data evinced that DM and poor glycemic control in non-DM subjects enhance glucose hypometabolism in AD-related regions [119]. Additionally, during the progression of monkeys from healthy to pre-DM and to T2D conditions, the brain moves into altered metabolism showing increased glucose and reduced AB levels in CSF, with increased AB aggregation in the brain, a transition consistent with the human AD progression [124]. Indeed, several reports have reinforced the association of IR/T2D with reduced 2-FDG uptake in AD-related regions [104,125]. Furthermore, a recent review depicted, in cognitively normal elderly individuals with obesity and/or DM diabetes, the IR-related loss of neurons and/or impairment of neuronal glucose utilization in specific brain areas [120].

Finally, as the reduced expression of GLUT4 in some peripheral tissues is a signature of peripheral IR, and that seems to also be involved in the reduction of BGU in AD-related areas, that is another important common pathophysiological mechanism of AD and T2D. This will be discussed below in Section 7.

### 5.3. BBB Dysfunction

DM is known to induce microvascular disease in several territories, and dementia has been related, among several other factors, to BBB dysfunction. To our understanding, in the association of DM with dementia, brain microvascular disease and BBB dysfunction should be treated as the same condition.

More than 30 years ago, AD was proposed to be related to DM-induced brain microangiopathy. AD was associated with vascular basement membrane (VBM) alterations of brain microvasculature, including both thickening and vacuolization, with the alteration of several VBM components (collagen type IV, laminin, and heparan sulfate proteoglycan (HSPG)), as observed in the kidneys of patients with DM [126]. Like the leakage of proteins in diabetic nephropathy, the BBB alteration in the brain has been associated with the anomalous flux of some molecules, such as albumin, pro-inflammatory cytokines like TNF and interleukin 6 (IL6), lipoproteins, vitamin C, dextran, and some amino acids like the branched-chain neutral amino acids, which may contribute to amyloid plaque-associated degenerating neurites [42,53,125,126].

Since then, DM microvascular disease has contributed to the recognition of DM as a risk factor mainly of VD; however, the mechanisms underlying this association are not completely understood [93]. The homology of cerebral and retinal microvasculature, probably due to their similar embryological origin, provides some insight that, like retinopathy, cerebral small vessel disease (SVD) should also develop in DM (for a review, see [127]). In fact, SVD in DM subjects, identified as silent brain infarcts in magnetic resonance imaging, is associated with cognitive decline depending upon the region infarcted; because of that, DM retinopathy has been considered as a marker for cognitive impairment in DM subjects [128]. On the other hand, microvascular damage/BBB dysfunction has also been related to cognitive decline in AD. Using contrast-enhanced MRIs of brains of aged humans, BBB breakdown in the hippocampus was correlated with mild cognitive impairment [117]. Indeed, BBB breakdown has been associated with hippocampal-dependent cognitive impairment in obese rats [129], aging humans [130], and obese aging humans [131].

A question that remains unanswered is the possible development of DM-related alterations in glucose transport across the BBB. In rats, it is well accepted that chronic hyperglycemia is accompanied by decreased BBB GLUT1 expression [42]; however, that is unknown in humans, where GLUT1 expression and distribution assume special patterns in different types and sections of brain capillaries (as commented on in Section 3.2). Moreover, the literature reports the participation of changes in SGLT1/2 expression [42]; however, as commented on above (Section 3.2) neither the expression of any SGLT isoform nor the Me-4FDG transport has been demonstrated in the human BBB.

Despite that gap in the knowledge regarding glucose flux through the BBB, the fact is that DM increases the CSF glucose concentration, indicating that the glucose flux is preserved, and the hyperglycemia-induced increase in the blood/tissue glucose gradient ensures a high glucose concentration in the interstitium and CSF. This means that all the reduction in BGU (hypometabolism), observed in the AD signature, should not be ascribed to any BBB-related impairment of glucose flux.

### 5.4. Common Biomarkers for DM and AD

T2D and AD share several common features, some of them commented on above. A recent systematic review has searched for matching peripheral proteomic biomarkers of AD and T2D; seventeen common biomarkers that were differentially expressed in both AD and T2D subjects were identified, as compared to healthy controls [132]. As proposed by the authors, those biomarkers reinforce the T2D/AD relationship, and could provide a useful workflow for screening T2D subjects at risk of developing AD [132].

## 6. AD, DM, and GLUT1-3 and SGLT1 in the Hippocampus

As presented above, the development and progression of cognitive impairment, such as that associated with AD, have been clearly related to reduced glucose utilization in specific brain areas. Among them is the hippocampus, which is one of the most important, because of its critical role in memory. Considering that, naturally, attention was drawn to the mechanisms of neuronal glucose supply in the hippocampus, and other AD-related areas as well, surely involving glucose transporters GLUT1-3 and SGLT1.

In 1993, Simpson and colleagues reported that in the brains of patients with AD, the amount of GLUT1 and GLUT3 was reduced in some regions, including the hippocampus; the reduction was larger for GLUT3, and was accompanied by a significant loss of synapses [133]. On the other hand, the GLUT1 analysis was greatly compromised by the fact that GLUT1 is widely expressed in brain cells, which were not discriminated in the analysis [133]. Furthermore, reduced GLUT3 expression was also described in the human neuronal cell line SH-SY5Y, challenged with high concentrations of insulin (100 nM) for 16 h [65].

An elegant study has analyzed the expression of GLUT1-3 in crude extracts of the frontal cerebral cortices of brains from control and AD subjects. Reduced (25–30%) expressions of GLUT1 and GLUT3 were observed in AD brains, with a great increase in GLUT2 [74]. The reduction in GLUT1 and GLUT3 correlated negatively to tau phosphorylation and to the density of neurofibrillary tangles, evincing that reduced GLUT1 and GLUT3 may contribute to hyperphosphorylation of tau and impaired neurofibrillary degeneration in AD [74]. However, we must consider that while GLUT3 reduction can be certainly ascribed to neurons, GLUT1 reduction may involve several cell types, especially endothelial cells and erythrocytes of the microvasculature, which express GLUT1 in high amounts. Moreover, since GLUT2 expression in neurons was described only in CVOs, the increase in GLUT2 in the cortex of AD subjects was ascribed to the activation of astrocytes. In fact, the glial fibrillary acid protein (astrocyte marker) was also increased in AD brains, confirming the astrocyte activation [74], and revealing a local pro-inflammatory activity in AD. Unfortunately, in this study there was no information regarding the metabolic state of the investigated subjects [74].

In addition to GLUT2 and GFAP, two other markers have been related to astrocyte activation in AD pathophysiology: the S100 calcium-binding protein B (S100-B) and JAK/STAT (janus kinase/signal transducer and activator of transcription) signaling. The S100B protein has been reported to exacerbate AD pathology, but the ability of S100B inhibitors to prevent/reverse AD injuries remains controversial. However, the knockout of the *Sb100* gene (PSAPP/*S100b*-/- mice) induces a reduction in cortical plaque load, gliosis, and neuronal dysfunction; therefore, that could be a novel strategy to fight AD injuries [134]. The participation of JAK/STAT signaling in the AD has been highlighted from the observation that leptin, by binding to its receptor, activates several pathways including JAK/STAT signaling in the hippocampus, revealing a protective role in AD pathophysiology [135]. Later, the JAK-STAT3 pathway merged as a central regulator of astrocyte activation by revealing that each specific functional state of reactivity was governed by complex molecular interactions within astrocytes, which converge on STAT3 [136]. Reviews concerning the AD-related markers of astrocyte activation and neuronal apoptosis are available elsewhere [137,138].

Regarding SGLT1, despite its presence (low amount) in neurons of the hippocampus, reports of SGLT1 alteration associated with pathological conditions are rare; increased SGLT1 expression was described as a consequence of seizures or traumatic brain injury (TBI) in experimental models, and also in the brain from an individual that died after TBI, as compared to those who died of cardiac arrest [39].

Alterations in glucose transporters and glucose metabolism in AD-related regions have directed attention to these fields, and several reviews have been published on this matter. However, some reviews have provided a minor contribution to the knowledge of the true role of glucose transporters in AD/diabetes association, sometimes unnecessarily discussing several glucose transporter isoforms not related to the AD-damaged regions and including unprecise concepts [42,63,139]. On the other hand, a good review has shed light on the role of brain glucose metabolism and cognitive dysfunction in DM and AD [118].

## 7. AD, DM, and GLUT4 in the Hippocampus

Altered GLUT4 expression in some specific areas of the brain, including the hippocampus and the cerebellum (Section 3.4), was reported in metabolically impaired animals such as hyperinsulinemic db/db mice and streptozotocin-diabetic rats [72]. The association of altered GLUT4 expression and metabolically-related cognitive dysfunction was firstly reported in obese Zucker rats (a model of T2D). Zucker rats were tested for learning and memory, and impaired performance dependent of the hippocampus was observed, accompanied by decreased plasma membrane GLUT4 expression in hippocampal neurons, without apparent changes in total GLUT4 content [140]. Moreover, in human neuronal SH-SY5Y cells exposed to a 16-h insulin (100 nM) treatment, an important reduction in GLUT4 expression, and loss of the ability of translocating in response to acute insulin stimulation, were observed [65]. These changes are similar to those observed in the classic insulin-sensitive tissues of obese T2D mice [141].

Recently, we have investigated the GLUT4 expression and possible AD-related mechanisms in postmortem brains from obese and obese + T2D subjects [76]. Hippocampal neurons of the *cornu ammonis* 4 area (CA4 area) from obese + T2D subjects displayed reduced GLUT4 expression and neuronal soma area, associated with an increased expression of NFKB-p65 (a marker of inflammatory activity). Interestingly, increased inflammatory activity (NFKB-p65 expression) was also observed in the CA4 area from obese subjects without T2D, although decreased GLUT4 expression and reduced neuronal soma area have not been detected in these subjects yet [76]. This observation indicates that inflammation is an important mechanism for neurodegeneration during the health–T2D transition. Additionally, no alterations in the immunoreactivity for carboxymethyllysine (a marker of AGE activity) was observed in hippocampal CA4 neurons from obese or obese diabetic subjects [76].

Moreover, in isolated human SH-SY5Y neurons, inflammatory activation induced by tumor necrosis factor (TNF) could reduce the expression of *SLC2A4*/GLUT4 and of some genes related to neuronal function (synaptophysin, synapsin-1, and tyrosine hydroxylase) and their respective proteins [76]. Moreover, TNF increased NFKB binding activity in the *SLC2A4* promoter (a powerful repressor of the *SLC2A4* gene [142]), explaining a transcriptional mechanism involved in the reduction of *SLC2A4* gene expression [76]. Furthermore, increased histone acetylation status, by treating neurons with RGFP-996 (a histone deacetylase 3 inhibitor), increased *SLC2A4* expression and the total neuronal content of CRE-binding proteins (CREB/ICER), and counterbalanced the TNF-induced repressor effects, revealing the additional participation of epigenetic mechanisms in these effects [76]. These results uncover the improvement of GLUT4 expression and/or inhibition of HDAC3 as promising therapeutic targets to fight DM-related neurodegeneration [76]. Finally, in response to 24-h treatment with AGE-albumin (compared to control albumin), SH-SY5Y cells also repressed *SLC2A4*/GLUT4, an effect not related to NFKB repression upon the *SLC2A* gene; furthermore, AGE-albumin did not alter the expression of synapsin-1, tyrosine hydroxylase, or synaptophysin (both mRNAs and proteins), indicating that, despite the reduction in the GLUT4 content and expected glucose uptake, some markers of neuronal function are still preserved [76].

The role of IR and GLUT4 in hippocampal-dependent memory has been strengthened in the last decade (for a review, see [24,81]). For that, several antidiabetic drugs have been evaluated in searching for a beneficial effect on the risk of dementia. These drugs include metformin, thiazolidinediones, pioglitazone, dipeptidyl peptidase-4 inhibitors, α-glucosidase inhibitors, meglitinides, insulin, sulphonylureas, glucagon-like peptide-1 receptor agonists (GLP1RAs), and sodium-glucose cotransporter-2 inhibitors (SGLT2is). From these, metformin, thiazolidinediones, pioglitazone, GLP1RAs, and SGLT2is were associated with reduced risk of dementia [143]. More longitudinal studies aimed at determining their relative benefit in different populations should be conducted. In this regard, considering the well-known insulin sensitizer role of metformin, which includes the improvement of GLUT4 expression/translocation in adipose and muscle tissues [144], we should direct attention to this promising drug to fight dementia. In the PubMed database, when searching for “metformin, Alzheimer and Review”, 103 occurrences are shown, with 16 only in 2023 (up to October). Most of these reviews, including some meta-analyses that only evaluated the risk of AD development progression, conclude that metformin decreases the risk of DM-related AD. Some other reviews have extended the analysis to the mechanisms possibly involved, but none have analyzed positive effects upon GLUT4 yet. A recent important review, evaluating protective mechanisms of metformin against DM-induced neurodegeneration, points out that metformin has beneficial effects against central inflammatory, mitochondrial, and oxidative stress, as well as anti-apoptotic effects [145].

## 8. Concluding Remarks

Since the 1960s, the association among DM, cognitive dysfunction, and brain damage has been reported to be affiliated with recurrent hypoglycemia with low brain glucose concentration, secondary to pharmacological treatments for DM, especially insulin therapy in DM subjects. Even today, the pursuit of an excellent glycemic control, avoiding recurrent hypoglycemia to preserve cognitive function, is a major challenge in the treatment of T1D.

**Figure 2:**
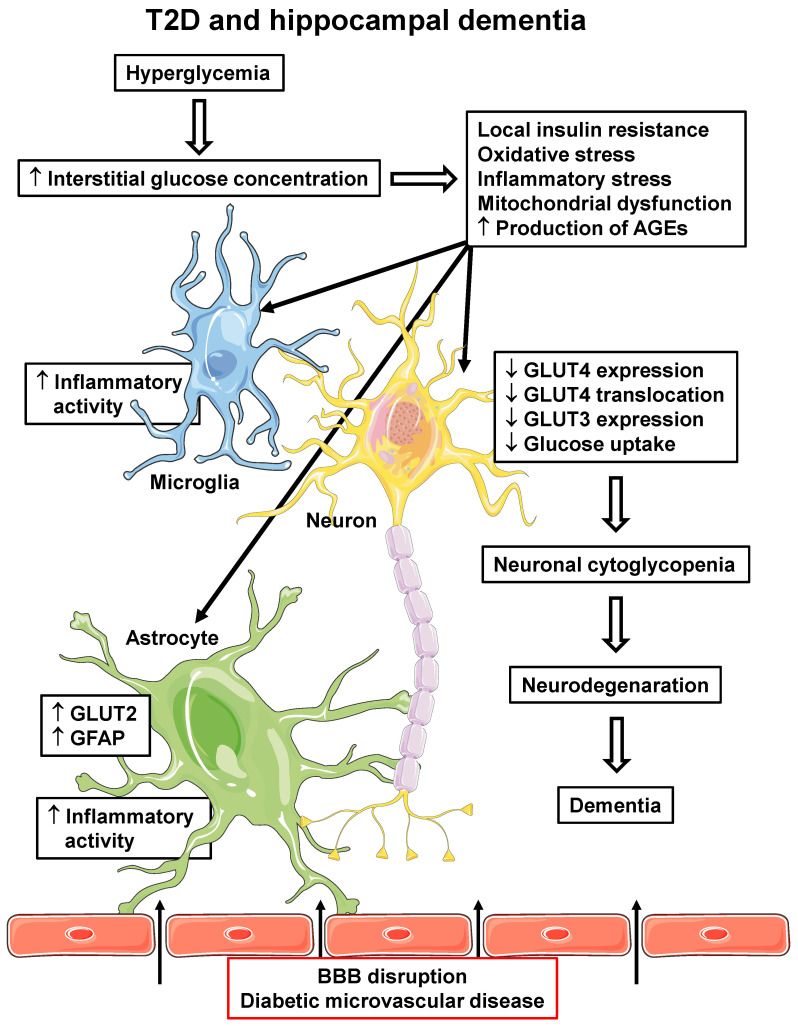
T2D and hippocampal dementia. T2D-related pathophysiological alterations in the hippocampus, which can participate in the development and/or progression of dementia. Hyperglycemia can lead to increased interstitial glucose concentration, which induces cellular insulin resistance, oxidative and inflammatory stress, mitochondrial dysfunction and increases AGE production, in all local cells. Increased microglia and astrocyte inflammatory activity reinforces local inflammatory activity; in astrocytes, increased GLUT2 and GFAP expression are related to pro-inflammatory activity. Additionally, diabetic microvascular disease (BBB disruption) promotes the leakage of some peripheral molecules into the hippocampal parenchyma (indicated by arrows), including some pro-inflammatory cytokines and AGEs, contributing to the reinforcement of their local generation. In neurons, alterations lead to decreased GLUT4 and GLUT3 expression and decreased PM translocation of GLUT4, eventually determining a decrease in cellular glucose uptake and metabolism. The final neuronal cytoglycopenia triggers cellular alterations that lead to neurodegeneration and dementia. AGEs, advanced glycated end products; BBB, blood–brain barrier; GFAP, glial fibrillary acid protein; GLUT2/3/4, facilitative glucose transporter type 2/3/4; T2D, type 2 diabetes mellitus. Parts of the figure were drawn using Servier Medical Art (https://smart.servier.com, accessed on 8 May 2023).

On the other hand, the increasing incidence of AD and T2D in the last 50 years has revealed a strong association between these two diseases, which seems to not be related to hypoglycemia. Indeed, peripheral IR, obesity, T2D, and AD are associated with brain hypometabolism and cognitive dysfunction, despite a high interstitial glucose concentration. Brain IR (especially in AD-related regions), together with inflammatory, oxidative, and mitochondrial stress, as well as increased AGE production, induce impaired GLUT4 translocation with further repression of the *SLC2A4* gene and GLUT4 protein expression in hippocampal neurons. That compromises neuronal glucose utilization, participating in the development/progression of hippocampal neuronal dysfunction and neurodegeneration. Figure 2 summarizes potential events involved in the development T2D-related hippocampal damage.

This knowledge should encourage further investigations into beneficial, promising therapeutic approaches, regarding central insulin sensitivity and GLUT4 expression, to fight diabetes- and Alzheimer-related neurodegeneration.

## Data Availability

Not applicable.

## Abbreviations

Abbreviations (and names) of genes and proteins are in accordance with the HGNC and UniProt databases, respectively.

2-FDG	2-[^18^F]fluoro-2-deoxy-D-glucose (substrate for GLUTs)
2-hPG	2-h post-load glucose
11C-PiB	[11C]-labeled Pittsburgh compound B (marker for amyloid beta accumulation)
AB	amyloid-beta
ABPP	amyloid beta precursor protein
AD	Alzheimer’s disease
AGE	advanced glycation end product
AMG	[^14^C]α-methyl D-glucopyranoside
AS160	AKT substrate 160
BAD	Bcl2-associated agonist of cell death
BBB	blood–brain barrier
BBMEC	bovine brain microvascular endothelial cell
BGU	brain glucose utilization
CA4	cornu ammonis 4 area
CSF	cerebrospinal fluid
CNS	central nervous system
CREB/ICER	CRE-binding proteins
DHA	dihydroxyacetone
DM	diabetes mellitus
FOXO1	forkhead box protein O1
GA	glycated albumin
GAPDH	glyceraldehyde-3-phosphate dehydrogenase
GLUT1–GLUT8	glucose transporter type 1–8 proteins
GSK3B	glycogen synthase kinase 3 beta
HbA1c	glycated hemoglobin A1c
hCMEC/D3	human cerebral microvascular endothelial cell line/D3
HOMA-IR	homeostatic model assessment for insulin resistance
IGF1	insulin-like growth factor 1
IL6	interleukin 6
IR	insulin resistance
JAK	janus kinase
*MAPT*	microtubule associated protein tau gene (human)
Me-4FDG	α-methyl-4-[18F]fluoro-4-deoxy-D-glucopyranoside (substrate for SGLTs)
MGO	methylglyoxal
MRI	magnetic resonance imaging
NFKB-p65	nuclear factor NF-kappa-B subunit p65
OGTT	oral glucose tolerance test
PET	positron emission tomography
PI3K	phosphatidylinositol 3-kinase
PIP3	phosphatidylinositol 3,4,5-trisphosphate
PKB	protein kinase B (alias AKT)
PM	plasma membrane
pO2	partial pressure of oxygen
RAGE	advanced glycation end product receptor
ROS	reactive oxygen species
S100-B	S100 calcium-binding protein B
*S100b*	S100b gene (mouse)
SGLT1–2	sodium/glucose cotransporter types 1–2
*Slc2a1–8*	solute carrier family 2 members 1–8 genes (mouse)
*SLC2A1–8*	solute carrier family 2 members 1–8 genes (human)
*Slc5a1–2*	solute carrier family 5 members 1–2 genes (mouse)
*SLC5A1–2*	solute carrier family 5 members 1–2 genes (human)
STAT	signal transducer and activator of transcription
SVD	small vessel disease
TD1	type 1 diabetes mellitus
T2D	type 2 diabetes mellitus
TBI	traumatic brain injury
TNF	tumor necrosis factor
VD	vascular dementia
